# Electrical Conductivity Performance of Predicted Modified Fibre Contact Model for Multi-Filler Polymer Composite

**DOI:** 10.3390/polym11091425

**Published:** 2019-08-30

**Authors:** Nabilah Afiqah Mohd Radzuan, Abu Bakar Sulong, David Hui, Anil Verma

**Affiliations:** 1 Centre for Materials Engineering and Smart Manufacturing (MERCU), Faculty of Engineering and Built Environment, Universiti Kebangsaan Malaysia, 43600 UKM Bangi, Selangor, Malaysia; 2 Department of Mechanical Engineering, University of New Orleans, New Orleans, LA 70148, USA; 3 Department of Chemical Engineering, Indian Institute of Technology Delhi, Hauz Khas, New Delhi-110 016, India

**Keywords:** hot pressing, composites, electrical conductivity, fuel cells

## Abstract

Polymer composites have been extensively fabricated given that they are well-fitted for a variety of applications, especially concerning their mechanical properties. However, inadequate outcomes, mainly regarding their electrical performance, have limited their significant potential. Hence, this study proposed the use of multiple fillers, with different geometries, in order to improve the electrical conductivity of a polymer composite. The fabricated composite was mixed, using the ball milling method, before being compressed by a hot press machine at 3 MPa for 10 min. The composite plate was then measured for both its in-plane and through-plane conductivities, which were 3.3 S/cm, and 0.79 S/cm, respectively. Furthermore, the experimental data were then verified using a predicted electrical conductivity model, known as a modified fibre contact model, which considered the manufacturing process, including the shear rate and flow rate. The study indicated that the predicted model had a significant trend and value, compared to the experimental model (0.65 S/cm for sample S1). The resultant fabricated composite materials were found to possess an excellent network formation, and good electrical conductivity for bipolar plate application, when applying compression pressure of 3 MPa for 10 min.

## 1. Introduction

Current news reports claim that thousands of people globally are being affected by climate change and being forced to migrate from their homes, as riverbeds begin to dry up and forming what is referred to as, the “Dry Corridor”. Over the past decade, researchers have been attempting to discover alternate mechanisms that can help reduce the impact of climate change [1,2]. These include the use of solar energy, wind energy, and fuel cells as alternative materials reduce the impact on the environment [3,4]. Other materials, which are commonly employed in renewable industries nowadays are polymer composites, given that they are recyclable, have high mechanical properties, possess electrical conductivity, and have excellent thermal properties [5,6,7]. As with fuel cell applications, these polymer composite materials are generally fabricated as bipolar plate components, which are applied in polymer electrolyte membrane fuel cells (PEMFCs) [8,9,10].

Hence, researchers have conducted extensive studies on polymer composites, in order to increase their performance and tailor their properties to meet their specific needs of applications. These studies were carried out by adding multiple conductive fillers into selected polymer resins, using adequate techniques, including filler mixing and manufacturing processes. Even though studies on multiple fillers have been conducted over the last five years, these have failed to identify an optimum material composition and a suitable processing technique, in order to obtain a high polymer composite performance [11,12,13]. This is mainly attributed to the unique characteristics and structure of the fillers that were used, given different filler shapes and sizes result in the formation of different electrical conductivity networks [14,15,16]. Hence, the filler selection is crucial. A secondary filler, which is smaller in size and different in shape, should have a localised function within the primary filler [10,11]. These will form a network to increase the electrical conductivity and enhance the mechanical strength of the composites.

Numerous studies have shown that the predicted electrical conductivity model of the composite materials can minimise the number of experiments to be conducted [17]. By adapting the mechanical model, Lux (1993) introduced four types of electrical conductivity models, namely the statistical percolation model, thermodynamic percolation model, geometrical percolation model, and the structure-oriented percolation model. These models have been modified by researchers to obtain a general effective media (GEM) model, which basically focuses on the material composition. Although, somehow, these models have burdened researchers in the prediction of electrical conductivity below the percolation threshold, even if using different sizes and shapes of fillers are used [11,18,19]. Hence, by adapting the conventional structure-oriented percolation model, researchers have developed a new modified fibre contact model, which is able to predict the electrical conductivity of the different types of fillers, at different orientations, as a polymer composite materials undergo a manufacturing process [17]. Therefore, this study proposes a newly-modified fibre contact model for multi-filler polymer composites by conducting experiments using fillers, with different geometries and sizes, to improve the network conductivity and enhance electrical conductivity performance, especially in-plane conductivity of polypropylene reinforced milled carbon fibre-graphene-synthetic graphite (PP/MCF/xGNP/SG) composite.

## 2. Methodology 

The conductive fillers, that were employed in this study, consisted of milled carbon fibre (MCF), synthetic graphite (SG), and exfoliated graphene nano-platelets (xGNP), while polypropylene (PP) functioned as the polymer resin. The primary filler, MCF grade CFP-7-50, that was obtained from Shenzhen Yataida High-Tech. Co. Ltd. had a resistivity of 14 µΩm, a filler diameter of 9 µm, an aspect ratio of 34, an average filler length of 300 µm, and a density of 1.75 g/cm^3^. Since the electrical conductivity of polymer composite materials is often low, the secondary fillers are often incorporated in the materials in order to enhance their overall electrical performance [20,21]. Synthetic graphite (SG) in flake-like shape geometry, with a particle size of 74 µm and surface area of 1.5 m^2^/g, was supplied by Asbury Carbon, New Jersey. While, the xGNP in the flake-like geometry shape that was provided by XG Sciences, Inc., USA had a filler diameter in the range between 2 µm and 15 µm, with a bulk density of 2.2 g/m^3^. The polypropylene matrix in powder form (grade Titan-600) was supplied by Goonvean Fibres Ltd. The polypropylene (PP) powder had an average size of 90 µm, density of 910 kg/m^3^, and melting index of 10 g/10 min at 160 °C.

The primary filler, MCF, was initially mixed with the secondary conductive filler (xGNP/SG) at a rotational speed of 1200 rpm for 60 s, using a mechanical mixer (RM-20-KIKA WERK model). The compositions of the secondary conductive filler (xGNP/SG) were set at 1 wt.% for xGNP while, 4 wt.% for SG as the compositions were confirmed by prior studies [22]. The precise composition of secondary fillers is crucial, as both fillers offer the same in geometrical shape, but with different dimension sizes. Studies have confirmed that incorporating different filler sizes would improve its conductivity network, which lifts its electrical conductivity [11,21]. This initial stage was crucial as it resulted in a homogenous composite material within the conductive filler, before the composite material was mixed with the polymer resin, polypropylene (PP), using a ball mill. A homogenous composite material was required to aid in the connectivity of the filler, in order to form a network and increase the electrical conductivity of the composite material [11,23]. Thus, the polypropylene reinforced the milled carbon fibre-graphene-synthetic graphite (PP/MCF/xGNP/SG) composite, which was mixed with stainless steel balls, having a diameter of 10 mm at a ball to powder ratio of 4:1, and at a rotational speed of 200 rpm for 60 min [11]. To develop a bipolar plate fabricated from conductive polymer composite materials, the composites mixture was then poured onto a coin-shaped compressed mould, having a diameter of 25 mm. The compression process was conducted using a hot press compression moulding machine, at a constant temperature of 200 °C. However, in order to study the electrical conductivity and resistivity of the conductive polymer composite materials, a series of experiments were conducted as various parameters, and listed in Table 1.

The characterisation of the polypropylene reinforced milled carbon fibre-graphene-synthetic graphite (PP/MCF/xGNP/SG) composite, was performed using an X-ray diffractometer (Model D8 Advance, Bruker AXS Germany), in order to identify the X-ray diffraction of the composite material, at a range of between 5° and 90°. The CuKα diffraction beam, having a wavelength (λ) of 1.542 nm was applied during the procedure at the cross section of each sample. The diffraction patterns were then analyzed and refined using the Rietveld refinement (X’Pert High Score Plus) software. The confirmed composite material was then analyzed using the thermogravimetric analysis (TGA) and differential scanning calorimetry (DSC) to determine the melting temperature and capability of the composite material to relate the high temperatures (120 °C to 200 °C) that were applied to each sample [8,24]. The micrograph image was analyzed using a Scanning Electron Microscope (SEM), Model Quanta FEI (Quanta 400F, Malaysia). These micrograph images (cross-section) were then analyzed using Microsoft Visio software to identify each of the filler’s angles that were developed. The experiment was performed using a Mettler Toledo machine, within a temperature range of 30 °C to 900 °C, and at a temperature rate of 20 °C/min in an atmosphere of nitrogen gas. In addition, the composite material was also tested for its resistivity and electrical conductivity, in order to determine its overall performance. However, since prior studies suggested that the electrical conductivity should be performed at both, in-plane and through-plane, hence, the in-plane conductivity was measured using a Jandel four-point probe and recorded by an RM3 test unit [11,21]. Meanwhile, the through-plane conductivity and resistivity were measured at a constant pressure of 0.7 MPa using a through-plane electrical conductivity tester, manufactured by ZBT in Duisburg, Germany [12,25]. In prior studies, the details of the in-plane, and through-plane, conductivity have been discussed in prior studies, as it involves standard equipment, used to measure electrical conductivity. The resistivity was measured in the range between 0 bar and 30 bar; and the measurement unit was maintained in order to ensure accuracy.

## 3. Prediction of Electrical Conductivity by Mathematical Model

Predicting the electrical conductivity of the polymer composite materials, has been extensively discussed, due to its potential in reducing experimental trials [17]. Previous studies have indicated that the general effective media (GEM) equation seems to be a promising mathematical model for the prediction of the electrical conductivity of multiple fillers [11,26]. Although, the model only considers constituent elements, namely, the conductive fillers and polymer resin, while overlooking other processing factors that influence the overall formation of the conductivity network. In contrast, some authors have adopted the modified fibre contact model equation, based on their manufacturing processes, namely, extrusion and compression moulding, in order to specify the predicted value [27]. Hence, this study highlighted the improvements made by adapting the established modified fibre contact model in a multiple conductive filler system, focusing on in-plane conductivity. The equation for this model considers the shear rate, γ and rotational speed, α of a composite material, during the manufacturing process, because these affect the structure of the composite material, as expressed in Equation (1):

(1) $$\begin{array}{l} \sigma_{c}=\sigma_{m}+\left[\frac{4}{\pi }{\left(\frac{d_{c}}{d}\right)}_{MCF}{\left(\frac{l}{d}\right)}_{MCF}\left(cos^{2}\theta \right)\left(\frac{\varphi_{overall}}{Q}\sigma_{f_{MCF}}\alpha X\right)\right]\\ \;\;\;\;\;\;\;+\left[\frac{4}{\pi }{\left(\frac{d_{c}}{d}\right)}_{xGNP}{\left(\frac{l}{d}\right)}_{xGNP}\left(cos^{2}\theta \right)\left(\frac{\varphi_{overall}}{Q}\sigma_{f_{xGNP}}\alpha X\right)\right]\\ \;\;\;\;\;\;\;+\left[\frac{4}{\pi }{\left(\frac{d_{c}}{d}\right)}_{SG}{\left(\frac{l}{d}\right)}_{SG}\left(cos^{2}\theta \right)\left(\frac{\varphi_{overall}}{Q}\sigma_{f_{SG}}\alpha X\right)\right] \end{array}$$

where the electrical conductivity of each element, namely, the composite material, matrix and filler, are denoted as $\sigma_{c},\sigma_{m},\sigma_{f}$, respectively. *X* is the function of the number of contacts denoted as 2.84, *d* is the diameter (9 μm), $d_{c}$ is the diameter of the filler contact (1 nm), *l* is the filler length (300 μm), φ is the volume faction, θ is the filler orientation angle, and α is the ball milling rotational speed (rev/sec). Meanwhile, *Q* is the volume flow rate of the overall composite material, which is defined as given in Equation (2):

(2) $$Q=\frac{volume,\;cm^{3}}{time,\;s}$$

**Figure 1:**
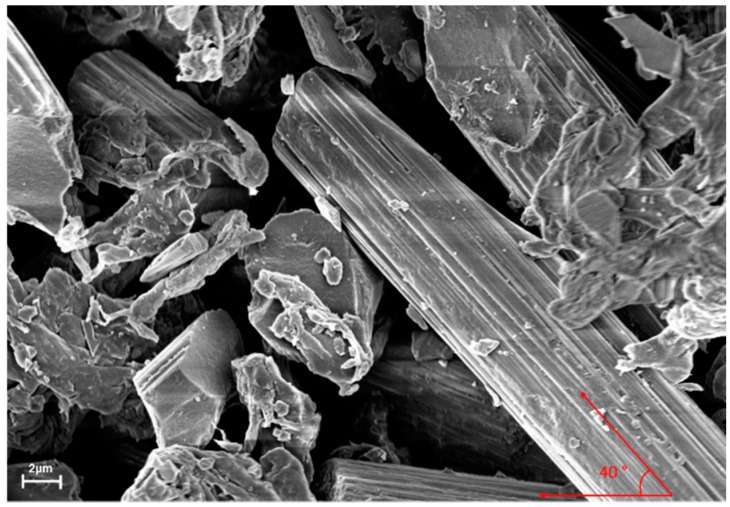
Orientation angle of carbon fibre at 40°.

The rotational speed of the ball milling machine was set at 3.33 rev/sec, while the processing time was considered in both, the pre-heated state, and during the compression moulding process. Also the conductivity of the polymer matrix, $\sigma_{m}$, which was estimated as 10^−17^ S/cm, was ignored as it was negligible and had no effect on the overall conductivity [10,26]. Meanwhile, *θ* in this study was standardised as 40°, based on the micrograph image of the composite materials, due to the inconsistent rotation of the fillers (Figure 1). This phenomenon arose from the breakage of the fillers, during the mixing process [28,29]. Hence, the simplified model showed the conductivity of the overall composite material, which consisted of primary filler and secondary fillers, as in Equation (3):

(3) $$\sigma_{c}=\left[\frac{4}{\pi }\left(\frac{d_{c}}{d}\right)\left(\frac{l}{d}\right)\left(cos^{2}\theta \right)\left(\frac{\varphi_{overall}}{Q}\sigma_{f}\alpha X\right)\right]$$

The electrical conductivity of the composite material, containing three different fillers using the specific manufacturing processes, was determined. 

## 4. Results and Discussion

### 4.1. Physical Characterisation of PP/MCF/xGNP/SG Composite

**Figure 2:**
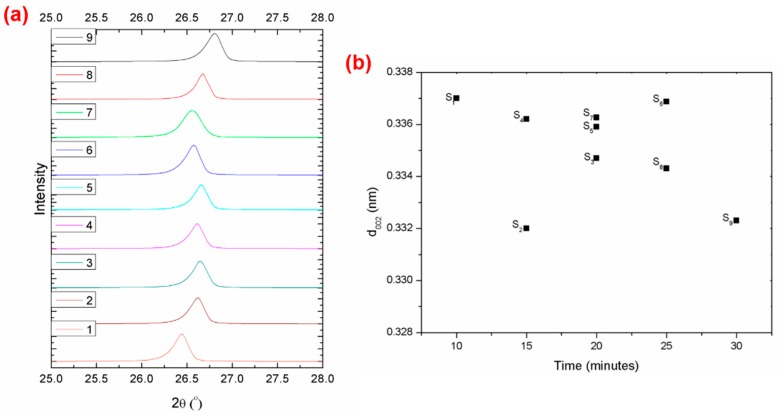
(**a**) XRD pattern of different composite compositions at 25° to 28°, and (**b**) changes in d_002_ at different processing times.

The XRD spectra of the elements of the composites materials showed the presence of pure carbon (C), due to the milled carbon fibre, graphene, and synthetic graphite that were applied in this study, as indicated in Figure 2a. Broad diffraction peaks could be clearly seen at around 26.4° to 26.6°, which were assigned to the (002) plane of the graphite structure, as confirmed by other related studies [30,31]. These diffraction peaks reported an average interlayer spacing (*d*_002_) of 0.33225 nm to 0.337 nm, as shown in Figure 2b. The *d*_002_ which remained constant, was identified as a graphitic structure, which was similarly reported by other studies [31,32,33]. Accordingly, this indicated that there was no drastic change in the structure of the composite during the compression moulding process [34]. Hence, it confirmed that the elements in the polypropylene reinforced milled carbon fibre-graphene-synthetic graphite (PP/MCF/xGNP/SG) composite had the same diffraction peaks and patterns as carbon, which was their main element. 

**Figure 3:**
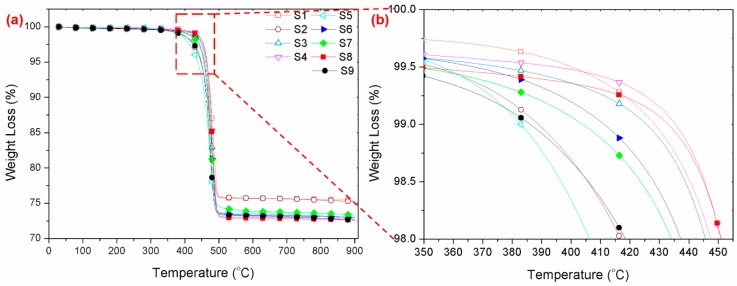
(**a**) Thermal degradation behaviour of polypropylene/milled carbon fibre/exfoliated graphene nano-platelets/synthetic graphite (PP/MCF/xGNP/SG) composite at different compression moulding parameters (**b**) Enlargement of Figure 3a.

A thermogravimetric analysis (TGA) was conducted, based on the XRD diffraction peaks. Figure 3a shows that the composite material experienced stable fluctuations in the range between 25 °C and 300 °C. Meanwhile, Figure 3b indicates that the composite material deteriorates severely at 385 °C and was fully decomposed at 500 °C, thereby showing an improvement of 28%, compared to conventional composites that decompose on average at 300 °C [8,12,35]. The samples, S_1_ and S_8_, which had been compressed at a pressure of 3 MPa, were able to withstand a maximum temperature of 385 °C without experiencing much loss in weight (~0.02%), compared to the other samples. In contrast, S_6_ experienced a drastic drop in weight (~0.06%) at a similar pressure, which might have been due to the prolonged compression time of 20 min, compared to S_1_ and S_8_ at 10 min, and 15 min, respectively. Studies have shown that an increase in compression time will result in unaligned fibre that weakens the composite structure and consequently, easily decompose it [36]. Although, as shown in Figure 3a, sample S_2_ demonstrates a higher remaining weight, as compared to the other samples. This might have occurred from insufficient bonding within the matrix; hence resulting in a separation between the polymer and the fillers, which further results in the fillers remaining and not being disposed of, unlike the other samples. These findings support the reasons for sample S_2_ to be obtained low through-plane conductivity (0.3 S/cm), given that the bonding within the filler was not robust enough, thereby resulting in weak electrons passing through the sample medium [37].

**Figure 4:**
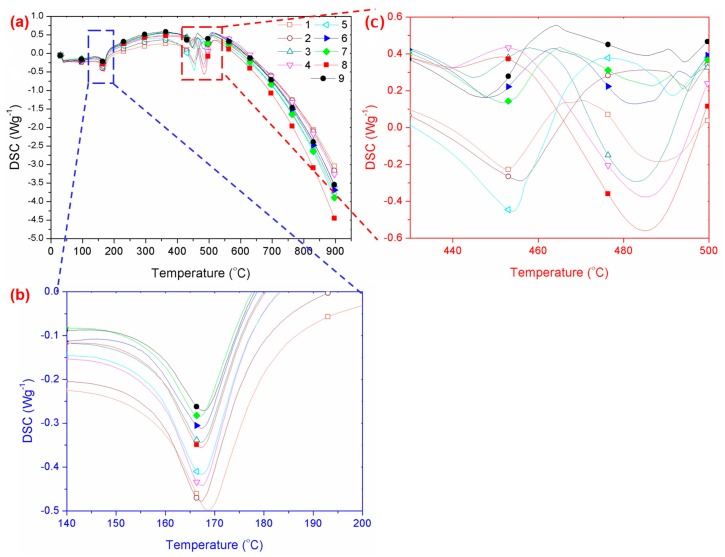
(**a**) Differential scanning calorimetry (DSC) curve of PP/MCF/xGNP/SG composite, as temperature reached 900 °C, (**b**) enlargement of (**a**) at temperatures ranging between 140 °C to 200 °C, and (**c**) enlargement of (**a**) at temperatures ranging from 430 °C to 500 °C.

Differential scanning calorimetric studies were conducted, in order to understand the effect of the composite properties on the melt temperature. Figure 4a represents the DSC curves of the PP/MCF/xGNP/SG composite at different processing parameters, with a baseline shift at ~200 °C and an endothermic peak at 455 °C. Based on the enlarged curve images displayed in Figure 4b, it can be seen that the composite encountered an earlier endothermic peak at ~168 °C, because the polypropylene resin started to melt [38]. The melting temperature of the polypropylene, used in this study, was slightly higher (~168 °C) because of its nano-sized powder, compared to standard polypropylene (~160 °C) [39]. Meanwhile, the second endothermic peak, as shown in Figure 4c, might have been due to the decomposition of the composite (multi-fillers). Hence, the melting temperature of the composite material was recorded at ~170 °C, even though the composite started to decompose at ~455 °C. However, there was no significant change in the overall melting temperature of the samples.

### 4.2. Electrical Conductivity Behaviour

**Figure 5:**
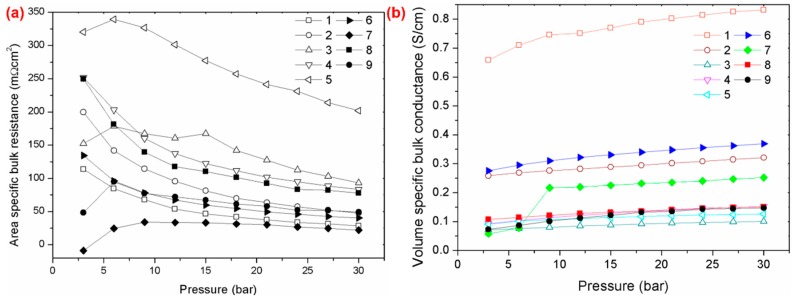
(**a**) Area specific bulk and (**b**) Volume specific bulk conductance resistance of PP/MCF/xGNP/SG composite at different pressures.

The conductivity of a polymer composite is often related to its electrical conductivity behaviour and resistivity as these affect the overall performance of the system. Figure 5a,b show the area specific bulk resistance and the volume specific bulk conductance of the polypropylene reinforced milled carbon fibre-graphene-synthetic graphite (PP/MCF/xGNP/SG) composite. The area-specific bulk resistance exhibited a significant decrease under pressure (untill 30 bar), thereby indicating that it was strongly dependent on the applied pressure [40,41]. Moreover, this phenomenon further caused an increase in electrical conductivity, as more pressure was applied to the composite masterials. Accordingly, this arose because, as the specific area decreases, the conductivity of the network often increases, with an increase in the contact areas [10,42]. This information comfirmed that the temperature, applied in this study, was the optimum temperature, as it had no effect on the resin used. Hence, the only connectivity that occurred within the matrix was between the filler chain links, compared to other composites that use an epoxy as the resin matrix [43,44]. These findings were supported, as shown in Figure 5b, where the volume specific bulk conductance increased, as more pressure was applied. Besides, sample S_1_ had the highest bulk conductance, compared to the other samples, due to its ability to achieve excellent electrical conductivity at a minimum compression time (10 min). Studies have suggested that this phenomenon is related to the nature of the filler itself, which is not perfectly straight [45]. Hence, a longer processing time will worsen and weaken the filler bonding, as the filler will be exposed to higher energy absorption. This will further result in an imperfect connection and connectivity within the filler, hence causing the overall electrical conductivity to deteriorate. This was proven in Figure 4b where the sample S_1_ encountered a lower energy absorption compared to the other samples.

**Figure 6:**
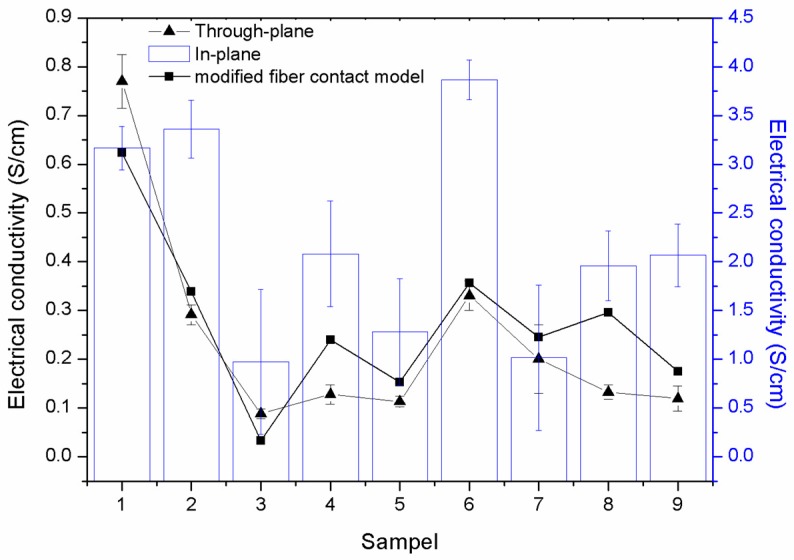
Conductivity (in-plane and through-plane) and predicted electrical conductivity model prediction using modified fibre contact model.

The electrical conductivity, especially of conductive polymer composites, fabricated as bipolar plates, is often measured in the in-plane, and through-plane, conductivities [11,46]. This is to ensure that the electrons are able to be transferred throughout the composite plates so as to produce excellent conductive materials. Figure 6 illustrates the in-plane and through-plane conductivities of the PP/MCF/xGNP/SG composite. Theoretically, the through-plane conductivity is often half the value of the in-plane conductivity [10,11,12]. This could be easily seen as all the samples recorded a through-plane conductivity were lower than the in-plane conductivity. Figure 6 shows that the samples S_6_, S_2_, and S_1_ displayed good in-plane conductivities of 3.9 S/cm, 3.5 S/cm, and 3.3 S/cm, respectively. The highest through-plane conductivity of 0.79 S/cm was recorded for sample S_1_, whereas the other samples had a lower through-plane conductivity. Therefore, based on this information, sample S_1_ appeared to have the most excellent electrical conductivity, as the electrons were able to pass through the composite material, yet have a good network formation within the composite material [21,47]. Notwithstanding this, various studies have suggested that the low electrical conductivity experienced by the samples, other than the S_1_ sample, was due to the fact that the composite material matrix encountered irregularities in the scattering fillers [43]. This phenomenon occurred as the composite material happened to undergo pressure for a longer compression time, which melted the polymer resin, as the melting temperature of polypropylene is 168 °C, as shown in Figure 4b. This consequently affected the fillers as they tended to orientate randomly, since there were no binders to bind them [48]. In addition, other studies have suggested that the motion of the electrons and the alignment of the fillers, within the composite material, are related to the movement of the charge carriers [43,49]. These support the lack of electrical conductivity at both, the in-plane, and through-plane, as the fillers tend to orientate randomly when the compression time and as pressure increases, thereby resulting in a poor conductivity network formation [50]. Accordingly, based on this information, we understand that, untill now, none of the studies was able to prove and fabricate composite materials with either, good electrical conducitivty, or excellent in mechanical properties. Moreover, these studies encountered significant effort in using multiple fillers at different filler loading and geometry sizes, in order to fabricate the most suitable composite materials for bipolar plate applications.

Prior studies have also indicated that electrical conductivity can be predicted using a mathematical model, based on statistical, thermodynamic, geometrical, and structured-oriented percolation models [51]. However, none of these models has been able to accurately predict the electrical conductivity of composite materials [16,18]. Additionally, extensive efforts have been made by researchers to predict electrical conductivity, with the latest proposal being a modified fibre contact model (Equation (3)). Figure 6 shows that the predicted model encountered a good trend in terms of the through-plane conductivity. Sample S_1_ recorded a maximum electrical conductivity of 0.63 S/cm, while a minimum electrical conductivity of 0.03 S/cm was recorded by sample S_3_. These predicted electrical conductivity values, for S_1_ and S_3_ values, demonstrated differential errors of 18%, and 62%, respectively, when compared to the experimental through-plane conductivity. Meanwhile, a minimum differential error of 6% was recorded for sample S_6_. This suggested that the predicted model is suitable for predicting the electrical conductivity of composite materials. However, a contrasting trend was shown when comparing the in-plane conductivities. This phenomenon was seen because the model that was applied only considered the filler orientation and the processing parameter (ball milling rotational speed).

## 5. Conclusions

This study managed to successfully fabricate a polypropylene reinforced milled carbon fibre-graphene-synthetic graphite (PP/MCF/xGNP/SG) composite (sample S_1_), at a compression pressure of 3 MPa, and a compression time of 10 min, as an alternate material for bipolar plates. The effects of different parameters in terms of the compression time and applied pressure on the temperature, resistivity, and electrical conductivity were observed. In-plane, and through-plane, conductivities of 3.3 S/cm, and 0.79 S/cm respectively were recorded. Although, there was a significant drop in the volume specific bulk conductance when a longer compression time was introduced, where S_1_ recorded a maximum of 0.8 S/cm, while the other samples recorded values in the range between 0.05 S/cm and 0.35 S/cm. The predicted data that was obtained was also significant when compared with the experimental through-plane results, which indicated that the predicted modified fibre contact model is suitable for predicting the electrical conductivity of composite materials. Moreover, these results demonstrated that the fabricated material is able to provide significant results which will be useful, especially in the fabrication of bipolar plates.

## Figures and Tables

**Table 1:** Parameters applied for polypropylene/milled carbon fibre/exfoliated graphene nano-platelets/synthetic graphite (PP/MCF/xGNP/SG) composite.

Sample	Preheat time (minutes)	Compress time (minutes)	Pressure (MPa)
S_1_	0	10	3
S_2_	0	15	5
S_3_	0	20	7
S_4_	5	10	5
S_5_	5	15	7
S_6_	5	20	3
S_7_	10	10	7
S_8_	10	15	3
S_9_	10	20	5
